# Biological target volume based on fluorine-18-fluorode-oxyglucose positron emission tomography/computed tomography imaging: a spurious proposition?

**DOI:** 10.1186/s13014-023-02225-4

**Published:** 2023-02-21

**Authors:** Ting Xu, Ye Feng, Huiling Hong, Yiying Xu, Jiawei Chen, Xiufang Qiu, Jianming Ding, Chaoxiong Huang, Li Li, Chuanben Chen, Zhaodong Fei

**Affiliations:** Department of Radiation Oncology, Clinical Oncology School of Fujian Medical University, Fujian Cancer Hospital, Fuma Road, Fuzhou, 350014 Fujian People’s Republic of China

**Keywords:** Nasopharyngeal carcinoma, ^18^F-FDG-PET/CT, Biological target volume, Local recurrence, Standardized uptake value

## Abstract

**Purpose:**

To assess whether the high metabolic region of fluorine-18-fluorode-oxyglucose (^18^F-FDG) in the primary lesion is the crux for recurrence in patients with nasopharyngeal carcinoma (NPC), to assess the feasibility and rationale for use of biological target volume (BTV) based on ^18^F-FDG positron emission tomography/computed tomography (^18^F-FDG-PET/CT).

**Methods:**

The retrospective study included 33 patients with NPC who underwent ^18^F-FDG-PET/CT at the time of initial diagnosis as well as the time of diagnosis of local recurrence. Paired ^18^F-FDG-PET/CT images for primary and recurrent lesion were matched by deformation coregistration method to determine the cross-failure rate between two lesions.

**Results:**

The median volume of the V_pri_ (primary tumor volume using the SUV thresholds of 2.5), the V_high_ (the volume of high FDG uptake using the SUV50%max isocontour), and the V_recur_ (the recurrent tumor volume using the SUV thresholds of 2.5) were 22.85, 5.57, and 9.98 cm^3^, respectively. The cross-failure rate of V_recur∩high_ showed that 82.82% (27/33) of local recurrent lesions had < 50% overlap volume with the region of high FDG uptake. The cross-failure rate of V_recur∩pri_ showed that 96.97% (32/33) of local recurrent lesions had > 20% overlap volume with the primary tumor lesions and the median cross rate was up to 71.74%.

**Conclusion:**

^18^F-FDG-PET/CT may be a powerful tool for automatic target volume delineation, but it may not be the optimal imaging modality for dose escalation radiotherapy based on applicable isocontour. The combination of other functional imaging could delineate the BTV more accurately.

## Introduction

Nasopharyngeal carcinoma (NPC) is a malignant head and neck cancer which is endemic in Southern China and Southeast Asia [1]. In the era of intensity-modulated radiotherapy (IMRT), definitive chemoradiotherapy remains the mainstay of treatment for NPC and results in excellent loco-regional control rates [2]. However, more than 10% patients develop local recurrence after primary treatment [3]. Aggressive salvage treatment for locally recurrent NPC (LR-NPC) may help achieve long-term survival [4]. However, salvage treatment for LR-NPC is complex and relatively limited [5]. Thus, the optimal solution for improving survival is to achieve adequate local control at primary treatment. One of the strategies for improving local control entails escalation of radiotherapy dose [6]. The bottleneck for this strategy is to determine the appropriate tumor volume to prescribe high radiation dose.

Traditional target volume delineation of nasopharyngeal tumors mainly relies on CT and MRI images, which are merely based on anatomical structure [7]. Functional imaging can help determine the biological characteristics and predict the radiotherapy sensitivity in different regions of the tumor; therefore, it has been gradually applied to contouring the gross target volume (GTV) [8, 9]. The development of functional imaging directly led to the emergence of the concepts of biological target volume (BTV) and biological IMRT. Fluorine-18-fluorode-oxyglucose positron emission tomography/computed tomography (^18^F-FDG-PET/CT) has evolved from a diagnostic and staging tool to a powerful modality for guiding cancer treatment in radiation oncology, especially for head and neck tumors [10, 11]. Previous studies have suggested that metabolic tumor volume (MTV) based on ^18^F-FDG-PET/CT can provide supporting information to target volume delineation for dose escalation [12, 13]. PET/CT-guided escalated dose painting, i.e., contouring the BTV in target volume has been proven to confer a considerable survival benefit for patients with LR-NPC [14]. However, there is a paucity of research exploring whether the recurrent lesion is indeed in the PET/CT-guided target volume, which may be supposed to benefit from receiving dose escalation.

Thus, this retrospective cohort study aimed to assess whether the lesion of local recurrence in patients with NPC highly coincides with the volume of high FDG uptake in the primary lesion. In other words, we sought to confirm whether the high metabolic region in the primary lesion is the crux for recurrence or not, so as to retrospectively assess the feasibility and rationale for BTV based on ^18^F-FDG-PET/CT. We used paired ^18^F-FDG-PET/CT imaging for primary and recurrent lesion matched by image coregistration method to determine the cross-failure rate between the two lesions of patients with LR-NPC.

## Methods and materials

### Patient selection

We retrospectively analyzed the medical records of patients with LR-NPC at our cancer center between January 2012 and December 2019. The inclusion criteria were: (1) biopsy-proven primary NPC; (2) confirmed diagnosis of locally recurrent NPC; (3) whole-body ^18^F-FDG-PET/CT conducted at the time of primary diagnosis and recurrent diagnosis; (4) received radical radiotherapy; (5) availability of complete medical records. The exclusion criteria were: (1) distant metastasis at first diagnosis; (2) second primary carcinoma; (3) severe medical complications; 4) disruption of treatment. Finally, a total of 33 patients were enrolled in the study. The ethics committee of the Fujian Cancer Hospital approved the study (no. YKT2020-011-01).

### ^18^F-FDG-PET/CT imaging

All patients underwent ^18^F-FDG-PET/CT on a Gemini TF 64 PET/CT scanner (Philips, The Netherlands) according to a previously published protocol [12]. Radiochemical purity of ^18^F-FDG exceeded 95% when manufactured by HM-10 cyclotrons. All patients fasted ≥ 6 h before undergoing ^18^F-FDG-PET/CT scanning. Serum blood glucose level was 3.9 to 6.5 mmol/L before ^18^F-FDG was intravenously administered at a dose of 148 to 296 MBq. Patients rested for 40 to 60 min in a dimly lit room before PET/CT scan. CT scanning was performed from the head to the proximal thigh and the scan slice thickness was 4 mm. An iterative reconstruction algorithm based on ordered-subset expectation maximization was used to reconstruct the PET images following the CT-based attenuation correction.

The FDG standardized uptake value (SUV) was measured based on the region of interest (ROI) of tumor, which was calculated as [(decay-corrected activity/tissue volume)/(injected dose/body weight)]. Primary tumors and recurrent lesion were delineated using the SUV thresholds of 2.5 according to previous studies [15, 16]. Maximum SUV (SUVmax) was defined as the value of the most intense voxel within the region of interest. The volume of high FDG uptake was defined within the primary lesion as the 50% isocontour of the SUVmax (SUV50%max).

### Treatment

All patients received radical IMRT. The radiotherapy dose and target volume delineation were conducted according to the institutional treatment protocol [17]. The GTV in the primary tumor (GTV-P) or in the involved lymph nodes (GTV-N) identified by clinical, imaging, and endoscopic results were prescribed to receive a total dose of 69.7–70.0 Gy administered in 33–35 fractions [17]. The planning target volume (PTV) was created with an additional 3 mm margin. Homogeneity Index (HI) is an objective tool for analyzing and quantifying the uniformity of dose distribution in the target volume. According to the International Commission on Radiation Units and Measurements (ICRU) report 83, the HI was calculated as $\frac{D2-D98}{D50}\times 100$ (D2, D98 & D50 are the doses to 2%, 98%, & 50% volume of PTV).

Patients with stage II-IV disease received concurrent chemoradiotherapy based on platinum with or without neoadjuvant chemotherapy, based on the institutional protocol [17].

### Follow-up and definition of recurrence

After treatment completion, follow-up was conducted once every 3 months for the first 2 years, once every 6 months in years 3 to 5, and annually thereafter until death or study end. Patients were assessed based on symptoms, physical examination, plasma Epstein-Barr virus (EBV)-DNA copy numbers, fiberoptic endoscopy, nasopharynx and neck contrast-enhanced MRI, chest CT, and abdominal ultrasound to monitor recurrence.

All patients with suspected local recurrence would receive fibreoptic endoscopy and biopsy. For patients without histological verification, the diagnosis of recurrence was confirmed by imaging signs of progression using ^18^F-FDG-PET/CT and MRI. The time to local recurrence was selected as the primary endpoint, defined as the duration from the date of diagnosis to the date of the first recurrence.

### Image coregistration and recurrence mode

**Fig. 1:**
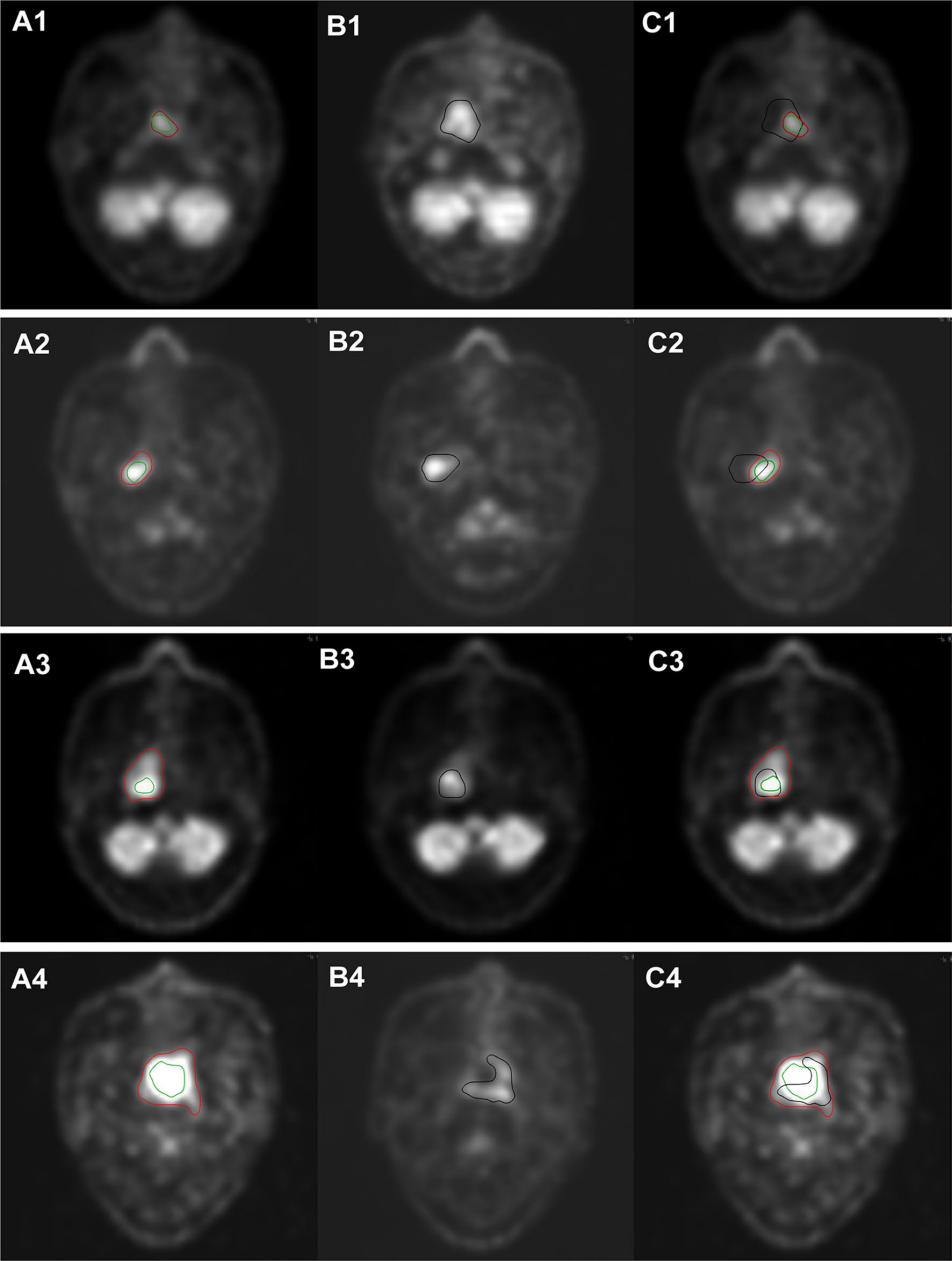
**A** ^18^F-FDG-PET/CT image at the time of primary diagnosis: the red line delineates the volumes with the SUV thresholds of 2.5 and the green line delineates the volumes with 50% of the SUVmax. **B** ^18^F-FDG-PET/CT image at the time of diagnosis of recurrence: the black line delineates the volumes with the SUV thresholds of 2.5. **C** The deformation coregistration ^18^F-FDG-PET/CT image of primary and recurrent images. (A1,B1,C1) Patient 1: Cross-failure rate of V_recur∩pri_ = 8.5%, out-field recurrence; Cross-failure rate of V_recur∩high_ = 6.29%, out-side recurrence. (A2,B2,C2) Patient 2: Cross-failure rate of V_recur∩pri_ = 47.76%, marginal-field recurrence; Cross-failure rate of V_recur∩high_ = 9.68%, out-side recurrence. (A3,B3,C3) Patient 3: Cross-failure rate of V_recur∩pri_ = 100%, in-field recurrence; Cross-failure rate of V_recur∩high_ = 66.73%, in-side recurrence. (A4,B4,C4) Patient 4: Cross-failure rate of V_recur∩pri_ = 97.15%, in-field recurrence; Cross-failure rate of V_recur∩high_ = 32.36%, marginal-side recurrence

^18^F-FDG-PET/CT images at the time of primary diagnosis and recurrent diagnosis were registered. For patients with LR-NPC, the primary tumor volume (V_pri_) was identified on primary ^18^F-FDG-PET/CT image (obtained at the time of diagnosis of primary NPC) using the SUV thresholds of 2.5 (Fig. 1-red line) [13]. The recurrent tumor volume (V_recur_) was identified on recurrent ^18^F-FDG-PET/CT image (obtained at the time when recurrence was first diagnosed) also using the SUV thresholds of 2.5 (Fig. 1-black line). The volume of high FDG uptake in primary tumor (V_high_) was defined on primary ^18^F-FDG-PET/CT image using the SUV50%max isocontour (Fig. 1-green line). SUV50%max target was selected according to the definition of BTV based on ^18^F-FDG-PET/CT [14]. As shown in Fig. 1, the volumes for the primary lesions (V_pri_), recurrent lesions (V_recur_) and high FDG uptake lesions (V_high_) were automatically delineated, respectively, using the AccuContour 3.2 software (www.manteiatech.com).

Using the deformation coregistration method, the ROIs in recurrent images were mapped to the primary images (Fig. 1C), and the volumes of the ROIs for recurrent and primary lesions were calculated. The exact site and extent of each recurrent tumor were then compared with the primary ^18^F-FDG-PET/CT image using V_pri_ and V_high_, respectively, focusing on the superimposition of V_recur_ to V_pri_ and V_high_. The overlap volume of V_recur_ and V_pri_ was defined as V_recur∩pri_, while the overlap volume of V_recur_ and V_high_ was defined as V_recur∩high_.

For the purpose of this study, the percentage of overlap for V_pri_ (SUV = 2.5) and V_high_ (SUV50%max) to V_recur_ (SUV = 2.5) was calculated. The cross-failure rate of V_recur∩pri_ was calculated as (V_recur∩pri_/V_recur_)*100%. Analogously, the cross-failure rate of V_recur∩high_ was calculated as (V_recur∩high_/V_recur_)*100%. The local recurrence was categorized as occurring inside or outside the V_pri_, depending on the position of V_recur_ and cross-failure rate of V_recur∩pri_: “in-field recurrence” if the rate was ≥ 95%, “marginal-field recurrence” if the rate was 20%–95%, or “out-field recurrence” if the rate was < 20%. Nevertheless, the cross-failure rate of V_recur∩high_ were delimited as: “in-side recurrence” if the rate was ≥ 50%, “marginal-side recurrence” if the rate was 20%–50%, or”out-side recurrence” if the rate was < 20% [18–20]. Representative ^18^F-FDG-PET/CT images of 4 patients was shown in Fig. 1 to better display each category of recurrence.

### Statistical analysis

Normality of distribution of continuous variables was assessed using the Shapiro–Wilk test. All normally distributed variables are expressed as mean ± standard deviation, whereas the skewed variables are expressed as median and range. The differences of SUVmax, V_recur_, V_pri_ and V_high_ between primary and recurrent ^18^F-FDG-PET/CT were evaluated using the paired *t* test (for normally distributed variables) or Wilcoxon signed-rank test (for skewed variables) (SPSS 22.0; IBM Corporation).

## Results

A total of 33 patients with LR-NPC qualified the inclusion criteria after a median follow up of 50 months (range 18–102). The clinical characteristics of the study population are summarized in Table 1. The average SUVmax of primary tumor was 11.43 ± 5.17, whereas the average SUVmax of recurrent lesions was 7.73 ± 4.06. V_pri_, the V_high_, and V_recur_ showed a skewed distribution, and the median volumes were 22.85 (range 4.16–105.09), 5.57 (0–43.07), and 9.98 (0.32–59.46) cm^3^, respectively.

**Table 1:** Baseline characteristics of 33 patients with locally recurrent NPC

Characteristic	Number of patients (%)
Sex	
Male	26 (78.79)
Female	7 (21.21)
Age (years)	
Mean ± SD	48.64 ± 10.29
≥ 50	14 (42.42)
< 50	19 (57.58)
Tumor category	
T1	5 (15.15)
T2	6 (18.18)
T3	11 (33.33)
T4	11 (33.33)
Node category	
N0	2 (6.06)
N1	13 (39.39)
N2	14 (42.42)
N3	4 (12.12)
Clinical stage	
II	3 (9.09)
III	15 (45.45)
IVa	15 (45.45)
SUVmax in primary tumor	
Mean ± SD	11.43 ± 5.17
SUVmax in recurrent tumor	
Mean ± SD	7.73 ± 4.06
V_pri_ in primary tumor (SUV = 2.5)	
Median (range)	22.85 (4.16–105.09) cm^3^
V_high_ in primary tumor (SUV50%max)	
Median (range)	5.57 (0–43.07) cm^3^
V_recur_ in recurrent tumor (SUV = 2.5)	
Median (range)	9.98 (0.32–59.46) cm^3^

*SD* Standard deviations, *SUV* Standardized uptake value, *V*_*pri*_ The primary tumor volume using the SUV thresholds of 2.5, *V*_*high*_ The volume of high FDG uptake using the SUV50%max isocontour, *V*_*recur*_ The recurrent tumor volume using the SUV thresholds of 2.5

The details of the TNM stage and dose distribution of the GTV-P for each of the enrolled patients are shown in Table 2. The median HI of the target volume was 0.07 (range 0.02–0.14), which indicated homogenous dose distribution among different radiotherapy plans. The GTV-P had sufficient dose coverage with only 0.39% (range 0–2.52) of the GTV-P obtaining < 95% of prescription dose. A greater part (95.74 ± 3.42%, range 87.98–100%) of GTV-P obtained ≥ 100% of prescription dose. The mean dose of GTV-P was 72.42 ± 0.89 Gy (range 69.34–74.16) while the minimum dose was 64.88 ± 4.12 Gy (range 55.09–69.38) and the maximum dose was 75.93 ± 1.69 Gy (range 71.45–78.38).

**Table 2:** Dose-volume histograms (DVHs) statistics for the gross tumor volume of primary tumor (GTV-P) in patients with locally recurrent NPC

No	Stage	HI	V95%	V100%	V110%	Dmax (Gy)	Dmean (Gy)	Dmin (Gy)
1	T2NT1M0 II	0.09	0.09	94.48	0.00	76.66	73.09	64.06
2	T2N2MO III	0.06	0.01	91.04	0.00	75.21	71.73	66.4
3	T1NT1M0 II	0.03	0.00	99.95	0.00	75.43	72.66	67.89
4	T3N1M0 III	0.09	0.62	95.72	0.00	76.81	72.98	62.95
5	T2N2M0 III	0.06	2.01	88.87	0.00	74.03	71.47	67.05
6	T4N1M0 IV	0.09	0.22	92.14	0.14	77.77	72.2	59.09
7	T4N2M0 IV	0.10	1.32	89.71	0.00	76.61	72.01	56.33
8	T1N2M0 III	0.08	0.01	91.02	0.00	75.82	71.99	66.42
9	T4N1M0 IV	0.09	0.11	97.30	0.68	78.38	73.19	64.48
10	T4N2M0 IV	0.14	2.52	87.98	0.21	77.74	72.73	62.57
11	T2NT0M0 II	0.09	0.02	95.01	0.00	76.19	73.08	66.25
12	T4N1M0 IV	0.09	0.25	97.09	0.00	76.97	73.34	55.09
13	T4N2M0 IV	0.05	0.25	98.55	0.00	76.64	72.69	55.71
14	T3N1M0 III	0.02	0.00	100.00	0.00	73.6	72.09	69.01
15	T3N2M0 III	0.05	0.04	95.55	0.00	74.31	72.19	65.56
16	T1N3M0 IV	0.03	0.00	98.01	0.00	72.69	71.4	69.38
17	T4N1M0 IV	0.04	0.07	98.71	0.00	75.08	71.38	61.7
18	T1N2MO III	0.10	0.21	95.82	0.92	77.83	73.01	65.13
19	T3N2MO III	0.07	0.00	95.75	0.02	77.18	73.17	63.8
20	T3N1M0 III	0.09	0.03	90.39	0.00	75.96	72.23	65.25
21	T3N1MO III	0.06	0.00	98.86	0.00	75.21	72.76	67.73
22	T2N2MO III	0.06	0.00	99.70	0.01	77.92	72.76	68.01
23	T3N3M0 IV	0.07	0.00	99.72	0.28	77.74	74.16	67.74
24	T2N2M0 III	0.08	0.24	94.03	0.00	75.86	72.48	57.93
25	T3N1M0 III	0.08	0.00	97.20	0.04	77.42	73.09	64.66
26	T3N3M0 IV	0.06	0.00	95.82	0.01	77.32	72.17	66.29
27	T3N2M0 III	0.02	0.00	96.90	0.00	72.45	70.85	67.01
28	T3N1M0 III	0.08	0.00	97.30	0.00	77.56	73.43	68.95
29	T1N3M0 IV	0.06	1.09	100.00	0.00	71.45	69.34	59.34
30	T4N2M0 IV	0.08	1.79	96.78	0.07	74.38	73.67	63.81
31	T4N1M0 IV	0.05	0.25	97.93	0.00	76.21	72.02	62.52
32	T4N2M0 IV	0.07	1.36	94.43	0.00	75.88	72.21	64.75
33	T4N0M0 IV	0.06	0.39	97.66	0.00	75.42	72.4	55.34

*HI* Homogeneity index, V95% = %Volume receiving < 95% of the prescribed dose, V100% = % Volume receiving > 100% of the prescribed dose; V110% = % volume receiving > 110% of the prescribed dose, *Dmax* Maximum dose, *Dmean* Mean dose, *Dmin* Minimum dose

The volume of ROIs (include V_pri_, V_high_ and V_recur_) and the cross-failure rate with corresponding recurrence mode for all patients are summarized in Table 3. The median percentage of V_high_ /V_pri_ was 34.14% (range 0–65.82).

**Table 3:** Details of the volume of ROIs and the cross failure rate with corresponding recurrence mode for all locally recurrent patients

No	V_pri_ (cm^3^)	V_high_ (cm^3^)	V_high_ /V_pri_ (%)	V_recur_ (cm^3^)	Cross-failure rate of V_recur∩pri_ (%)	Mode for V_recur∩pri_	Cross-failure rate of V_recur∩high_ (%)	Mode for V_recur∩high_
1	10.5	5.57	53.05	4.35	51.49	Marginal	26.44	Marginal
2	4.42	2.75	62.22	20.35	8.5	Out-field	6.29	Out-side
3	14.53	5.7	39.23	1.02	87.84	Marginal	37.65	Marginal
4	12.48	4.22	33.81	18.24	40.68	Marginal	20.72	Marginal
5	4.16	0	0	1.02	81.37	Marginal	0	Out-side
6	25.15	5.18	20.6	17.15	47.76	Marginal	9.68	Out-side
7	105.09	43.07	40.98	59.39	82.98	Marginal	40.09	Marginal
8	13.12	3.01	22.94	1.15	60.87	Marginal	22.61	Marginal
9	73.22	28.67	39.16	47.81	71.74	Marginal	18.74	Out-side
10	60.86	22.27	36.59	4.8	42.67	Marginal	4	Out-side
11	5.63	1.98	35.17	10.24	43.16	Marginal	18.16	Out-side
12	22.85	15.04	65.82	24.58	40.11	Marginal	30.19	Marginal
13	76.54	15.3	19.99	59.46	64.26	Marginal	17.12	Out-side
14	29.95	13.06	43.61	44.54	43.69	Marginal	23.42	Marginal
15	9.54	1.67	17.51	28.22	30.62	Marginal	5.92	Out-side
16	4.8	2.05	42.71	0.51	62.75	Marginal	25.49	Marginal
17	42.3	3.26	7.71	3.97	98.24	In-field	22.67	Marginal
18	9.47	4.1	43.29	0.9	77.78	Marginal	49.78	Marginal
19	11.65	3.58	30.73	8.06	73.08	Marginal	36.48	Marginal
20	45.57	6.98	15.32	0.704	100	In-field	18.47	Out-side
21	35.9	6.46	17.99	15.42	97.15	In-field	32.36	Marginal
22	7.3	0	0	11.71	51.41	Marginal	0	Out-side
23	37.76	10.5	27.81	13.44	74.78	Marginal	19.49	Out-side
24	9.28	1.98	21.34	1.09	100	In-field	64.22	In-side
25	29.31	3.804	12.98	6.27	96.97	In-field	62.2	in-side
26	8.26	2.82	34.14	4.03	98.51	In-field	60.3	in-side
27	44.67	11.9	26.64	6.21	86.63	Marginal	1.03	out-side
28	33.86	11.33	33.46	48.06	52.33	Marginal	21.31	marginal
29	5.57	2.3	41.29	0.32	100	In-field	81.25	In-side
30	60.86	22.14	36.38	9.98	100	In-field	66.73	In-side
31	53.7	23.62	43.99	6.08	29.44	Marginal	0	Out-side
32	57.34	28.22	49.22	21.5	82.74	Marginal	53.3	In-side
33	19.39	10.94	56.42	27.78	47.01	Marginal	29.01	Marginal

*V*_*pri*_ The primary tumor volume using the SUV thresholds of 2.5, *V*_*high*_ The volume of high FDG uptake using the SUV50%max isocontour, *V*_*recur*_ The recurrent tumor volume using the SUV thresholds of 2.5

**Fig. 2:**
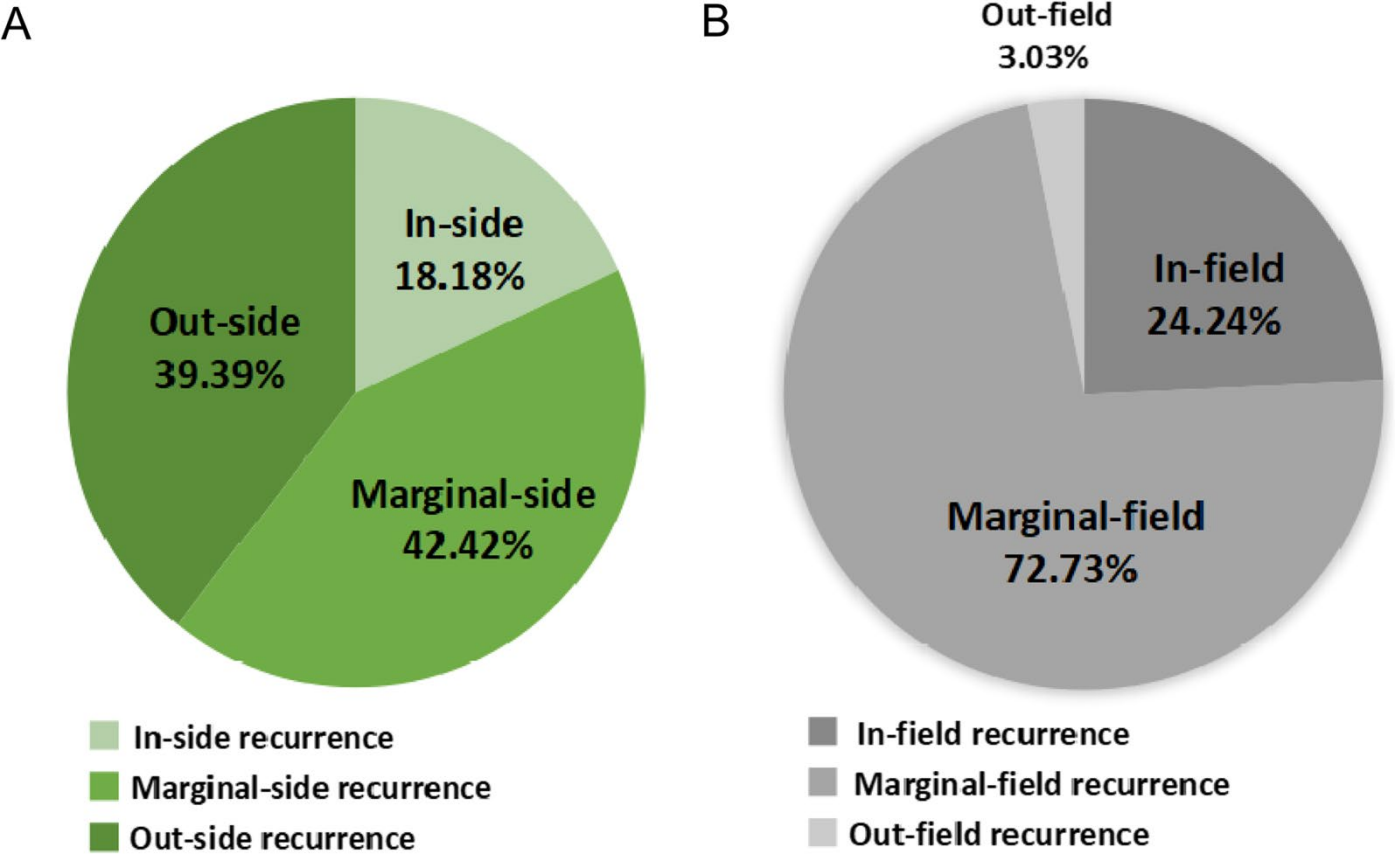
Pie chart of the cross-failure rate of V_recur∩high_ and V_recur∩pri_. **A** Cross-failure rate of V_recur∩high_; **B** Cross-failure rate of V_recur∩pri_

Based on the cross-failure rate of V_recur∩high_, the in-side, marginal-side, and out-side recurrence rates were 18.18% (6/33), 42.42% (14/33), and 39.39% (13/33), respectively (Fig. 2A). In other words, approximately 82.82% (27/33) of local recurrent lesions had less than 50% (marginal-side and out-side) overlap volume with the region of high FDG uptake in primary lesions.

As for the cross-failure rate of V_recur∩pri_, the in-field, marginal-field, and out-field recurrence rates accounted for 24.24% (8/33), 72.72% (24/33), and 3.03% (1/33), respectively (Fig. 2B). Approximately 96.97% (32/33) of local recurrent lesions had more than 20% (in-field and marginal-field) overlap volume with the primary tumor lesions. In addition, the median cross rate of V_recur∩pri_ was up to 71.74%.

## Discussion

Despite the application of IMRT and advances in treatment technology, local recurrence occurs in some patients with NPC, especially those with advanced disease. The reported local failure rate is up to 15–45% for T4 NPC, which remains an intractable problem [21, 22]. In comparison to the comprehensive treatment strategy involving chemotherapy, immunotherapy, and targeted therapy for patients with metastatic NPC, nasopharyngectomy and reirradiation remain the mainstay of salvage treatment for LR-NPC [21]. However, surgical salvage is only appropriate for early-stage recurrence. As for advanced LR-NPC, reirradiation is still the main treatment option. However, both surgery and reirradiation are associated with a high risk of early or late complications. Thus, exploring the mechanism of radiation resistance and improving the local control rate for primary NPC are key imperatives.

NPC is a highly radiosensitive and radiation dose-dependent disease, and the local control rate increases with improvement of irradiation dose, which is explained by a robust dose-tumor-control relationship [6, 23]. IMRT was shown to improve the dose and conformity of dose distributions in tumor volume by intensity-modulated radiation beams and iterative treatment plan optimization, which resulted in improved local control [24, 25]. However, due to the heterogeneity of biological characteristics and intratumoral radiation resistance, uniform radiotherapy dose may not kill all tumor cells. The inverse treatment planning system of IMRT enables the delivery of a small volume dose boost to target sites in tumor to increase the biologically effective dose (BED) [26]. Thus, identifying the region of high biological activity and resistance is the key to realize biology-guided radiotherapy (BgRT).

^18^F-FDG-PET/CT imaging can provide compositive information regarding anatomic site, receptor status, and metabolic processes. Currently, ^18^F-FDG is the most widely used radiopharmaceutical for PET, and its biological behavior is similar to that of glucose. One of the most distinctive biochemical features of malignant tumor cells is the high but inefficient metabolism of glucose, resulting in increased glycolysis [27]. FDG-6-PO4, the end product of FDG trapped in the metabolizing tissues, is a quantitative marker of the rate of glycolysis in the tumor. Compared with other positron-emitting radiotracers, FDG offers distinct advantages of long half-life and automated radiolabeling [27, 28]. In addition, Pisani et al. showed that the recurrence of head and neck carcinoma may originate from the volume with the highest FDG-signal [29]. Up to now, ^18^F-FDG-PET/CT has been used to identify biological target volumes for planning of radiation dose in non-small-cell lung cancer (NSCLC) and head and neck cancer (HNC) [10, 30].

In a prospective trial, PET/CT was shown to enhance the precision of the radiation therapy target volume definition using the SUV of 2.5 or greater (threshold value of 40%) as compared to CT in NSCLC [30–32]. Analogously, Sonay et al. assert that PET/CT can provide benefits in assessed target tumor volume for radiotherapy planning in patients with HNC [33]. In the present study, we also found that the SUV of 2.5 can reasonably guide the contouring of the GTV. Our results show that most of the local recurrent lesions had acceptable overlap volume (the median cross rate of V_recur∩pri_ was up to 71.74%) with the primary tumor lesions at the SUV of 2.5. Therefore, ^18^F-FDG-PET/CT can be considered as a powerful tool for automatic target volume delineation.

The ultimate goal of dose escalation radiotherapy is to improve the local control rate and overcome radiation resistance. The most direct manifestation of radiation resistance is recurrence. Therefore, we infer that the location of local recurrence lesion can be used to define the delineation of the radiotherapy target volume with high biological activity and radiation resistance. Functional imaging modalities such as PET can be more intuitive than structural imaging in reflecting the region of biological malignancy, which also reflect the relationship of metabolism and radiation resistance. Thus, several recent studies have focused on PET/CT-guided dose escalation to delineate the BTV. For the treatment of locally advanced NPC, a randomized trial showed that PET/CT-guided dose escalation radiotherapy is well-tolerated with the SUV of 2.5 and appears to be superior to CT-guided chemoradiotherapy in volume delineation of the biological gross tumor [13]. In addition, in a retrospective study by Liu et al., PET/CT-guided dose-painting IMRT within the isocontour of SUV50%max was found to confer a significant survival benefit in patients with locoregionally advanced NPC [14].

In this study, we chose SUV50%max as the biological target volume because several ongoing clinical trials use the SUV50%max isocontour for dose painting [34, 35]. However, we found that adopting the SUV50%max for dose escalation radiotherapy is inappropriate, because only 18.18% (6/33) patients with LR-NPC showed more than 50% overlap volume with the region of high FDG uptake. We speculate that the treatment failure, especially local recurrence, may be attributable to the persistence of dormant tumor cells and residual tumor cells after local therapy [36]. While the dormant tumor cells are not exactly the region of high FDG uptake, the region of SUV50%max isocontour for dose escalation was not consistent with the local recurrent lesion in our research. In addition, functional imaging modalities include ^18^F-FDG-PET/CT and MRI-based functional imaging techniques such as diffusion-weighted imaging (DWI) and intravoxel incoherent motion DWI (IVIM-DWI) [7]. PET and DWI are both potential candidates for determining the BTV within the GTV for dose painting and escalation in HNC. For DWI, a low apparent diffusion coefficient (ADC) value indicated tumor presence, so that it was also arranged as a candidate for dose painting [37]. However, Antonetta et al. found that ^18^F-FDG-PET/CT and DWI provide different functional information, resulting in different biological targets with less overlap [38]. Thus, we believe that ^18^F-FDG-PET/CT may not be the optimal imaging for dose escalation radiotherapy.

Some limitations of the present study should be acknowledged. Firstly, this was a single-center retrospective study with a small sample size, which may have introduced an element of bias. Secondly, due to the difference of posture between the ^18^F-FDG-PET/CT examine images and the treatment position, the dose of GTV and possible bias in coregistration could not be directly evaluated; thus the inhomogeneity of the target was ignored. Further prospective trials are required to assess the discrepancy among various functional imaging-guided dose escalation strategies.

## Conclusion

Our results suggest that ^18^F-FDG-PET/CT may be a powerful tool for automatic target volume delineation, but it may not be the optimal imaging modality for dose escalation radiotherapy based on applicable isocontour. The combination of other functional imaging could delineate the biological target volume more accurately.

## Data Availability

Data are available upon reasonable request. The data sets generated during and/or analyzed during the current study are available from the corresponding author on reasonable request.

## Abbreviations

NPC: Nasopharyngeal carcinoma
IMRT: Intensity-modulated radiotherapy
LR-NPC: Locally recurrent NPC
GTV: Gross target volume
BTV: Biological target volume
^18^F-FDG-PET/CT: Fluorine-18-fluorode-oxyglucose positron emission tomography/computed tomography
MTV: Metabolic tumor volume
SUV: Standardized uptake value
ROI: Region of interest
PTV: Planning target volume
HI: Homogeneity Index
ICRU: International commission on radiation units and measurements
EBV: Epstein-Barr virus
BED: Biologically effective dose
BgRT: Biology-guided radiotherapy
NSCLC: Non-small-cell lung cancer
HNC: Head and neck cancer
DWI: Diffusion-weighted imaging
ADC: Apparent diffusion coefficient
